# Effects of speech duration and voice volume on the respiratory aerosol particle concentration

**DOI:** 10.1265/ehpm.24-00251

**Published:** 2025-03-05

**Authors:** Tomoki Takano, Yiming Xiang, Masayuki Ogata, Yoshihide Yamamoto, Satoshi Hori, Shin-ichi Tanabe

**Affiliations:** 1 Department of Architecture, Waseda University, 3-4-1 Okubo, Shinjuku, Tokyo 169-8555, Japan; 2 Department of Architecture, Tokyo Metropolitan University, 1-1 Minamiosawa, Hachioji, Tokyo 192-0397, Japan; 3 Architecture Course, Tokyo Polytechnic University, 5-45-1 Iiyamaminami, Atsugi, Kanagawa 243-0297, Japan; 4 Department of Infection Control Science, Juntendo University, Graduate School of Medicine, 2-1-1 Hongo, Bunkyo, Tokyo 113-8421, Japan

**Keywords:** Aerosol transmission, COVID-19, Speech duration, Super-emitter, Voice volume

## Abstract

**Background:**

SARS-CoV-2 (COVID-19) is transmitted via infectious respiratory particles. Infectious respiratory particles are released when an infected person breathes, coughs, or speaks. Several studies have measured respiratory particle concentrations through focusing on activities such as breathing, coughing, and short speech. However, few studies have investigated the effect of speech duration.

**Methods:**

This study aimed to clarify the effects of speech duration and volume on the respiratory particle concentration. Study participants were requested to speak at three voice volumes across five speech durations, generating 15 speech patterns. Participants spoke inside a clean booth where particle concentrations and voice volumes were measured and analyzed during speech.

**Results:**

Our findings suggest that as speech duration increased, the aerosol number concentration also increased. Through focusing on individual differences, we considered there might be super-emitters who emit more aerosol particles than the average human. Two participants were identified as statistical outliers (aerosol number concentration, n = 1; mass concentration, n = 1).

**Conclusions:**

Considering speech duration may improve our understanding of respiratory particle concentration dynamics. Two participants were identified as potential super-emitters.

**Supplementary information:**

The online version contains supplementary material available at https://doi.org/10.1265/ehpm.24-00251.

## Background

SARS-CoV-2 is transmitted through air via infectious respiratory particles. Transmission can occur not only through direct deposition in a short range from an infectious person but also through inhalation at both short and long distances. This occurs when infectious respiratory particles are released and spread to others during respiratory activities such as coughing, breathing, or speaking by an infected person [1–3]. The greatest amount of virus is released by a person infected with COVID-19 between the time of infection and the onset of symptoms, which may allow the infection to spread even before an infected person is aware of any symptoms [4]. Moreover, COVID-19 transmission not only occurs through coughing and sneezing but also through respiratory activities such as breathing and talking.

To determine effective measures for preventing infection via aerosol particles, it is necessary to identify the quantity and size distribution of aerosol particles emitted during respiratory activities. Morawska et al. [5] used an aerodynamic particle sizer (APS) to investigate the aerosol particle concentration and size distribution during respiratory activities such as breathing, coughing, and speaking. The experimental results showed that continuous-speech aerosol particle concentrations were higher than those during coughing. It has also been suggested that the movements of the vocal cords, lips, and tongue generate aerosol particles of different diameters.

Focusing on voice amplitude and phonemes, Asadi et al. [6, 7] asked participants to speak monosyllabic and bisyllabic words and short sentences at different voice volumes and compared the participants’ aerosol particle emissions. The APS measured particle emissions during speech in a clean air environment in a laminar flow hood. They reported a positive correlation between voice amplitude and particle emissions and that particle emissions differed for each phoneme.

Gregson et al. [8] examined aerosol particle concentrations in professional singers who were asked to breathe, cough, speak, and sing “Happy Birthday.” They showed that the concentration of aerosol particles resulting from singing was significantly higher than that when speaking and that the concentration of aerosol particles increased with increasing voice volume.

While many studies have focused on monosyllables and short sentences, actual speech duration ranges from short utterances, such as greetings, to conversations lasting several tens of seconds. However, only a few studies have used speech duration as a variable.

Asadi et al. [6] focused on individual differences. When particle emissions were compared experimentally between participants, some participants had an individual particle emission rate that exceeded the group mean by one standard deviation or more, and these participants were defined as super-emitters. They also reported the existence of both super-emitters with significantly higher particle emissions from breathing and with significantly higher particle emissions from speaking.

Ahmed et al. [9] defined a super-emitter as a participant whose particle emissions were statistical outliers among all participants’ data. After repeating a 5-s speech experiment focusing on the frequency of voice, one of 40 participants was considered a super-emitter.

Super-emitters may be responsible for super-spreading because a small number of infected individuals can cause multiple secondary infections. Therefore, an analysis of super-emitters should be included in studies on respiratory aerosol particle emissions.

This study aimed to clarify the effects of speech duration and volume on respiratory aerosol particle concentrations. Aerosol particles can be released not only during speech involving phonemes, but also by the movement of the vocal cords, lips, and tongue between phonemes in a sentence. Therefore, based on the hypothesis that as speech duration increases, the concentration of aerosol particles emitted from the mouth also increases, we conducted an experiment in which speech duration and voice volume were considered as variables.

## Methods

### Study design and data collection

The experiment was conducted with 11 participants in their early 20’s (males, n = 4; females, n = 7) over five days, from October 5 to October 9, 2022, in a laboratory at Waseda University. The participants were asked to speak for five different durations at three voice volumes for a total of 15 speech patterns (Table 1). To compare with longer speech duration than those in previous studies, we selected 60 s as the longest speech duration and compared a total of five patterns: 1 s, 5 s, 10 s, 30 s, and 60 s. Aerosol particle concentration, with diameters ranging from 0.3 to 10 µm, was measured using an optical particle sizer (OPS, TSI: OPS3330) while the participants spoke. The OPS measurement particle-size range can be freely subdivided into 16 channels, as shown in Additional file 1. Voice volume during speech was simultaneously measured using a sound-level meter (RION: NL-42A) placed in front of the mouth. As it was difficult for the participants to speak continuously at a specific volume, they were asked to speak in three patterns at their discretion: loud, medium, and quiet.

**Table 1:** Speech patterns

**No.**	**1**	**2**	**3**	**4**	**5**
Speech duration (s)	1	5	10	30	60
Voice volume	Medium				

**No.**	**6**	**7**	**8**	**9**	**10**
Speech duration (s)	1	5	10	30	60
Voice volume	Loud				

**No.**	**11**	**12**	**13**	**14**	**15**
Speech duration (s)	1	5	10	30	60
Voice volume	Quiet				

Participants were asked to speak five sentences of different lengths at three voice volumes, for a total of 15 speech patterns. The experiment was performed in the following order: the first five patterns were at medium voice volume, the next five patterns at loud volume, and the last five patterns at quiet volume.

**Fig. 1:**
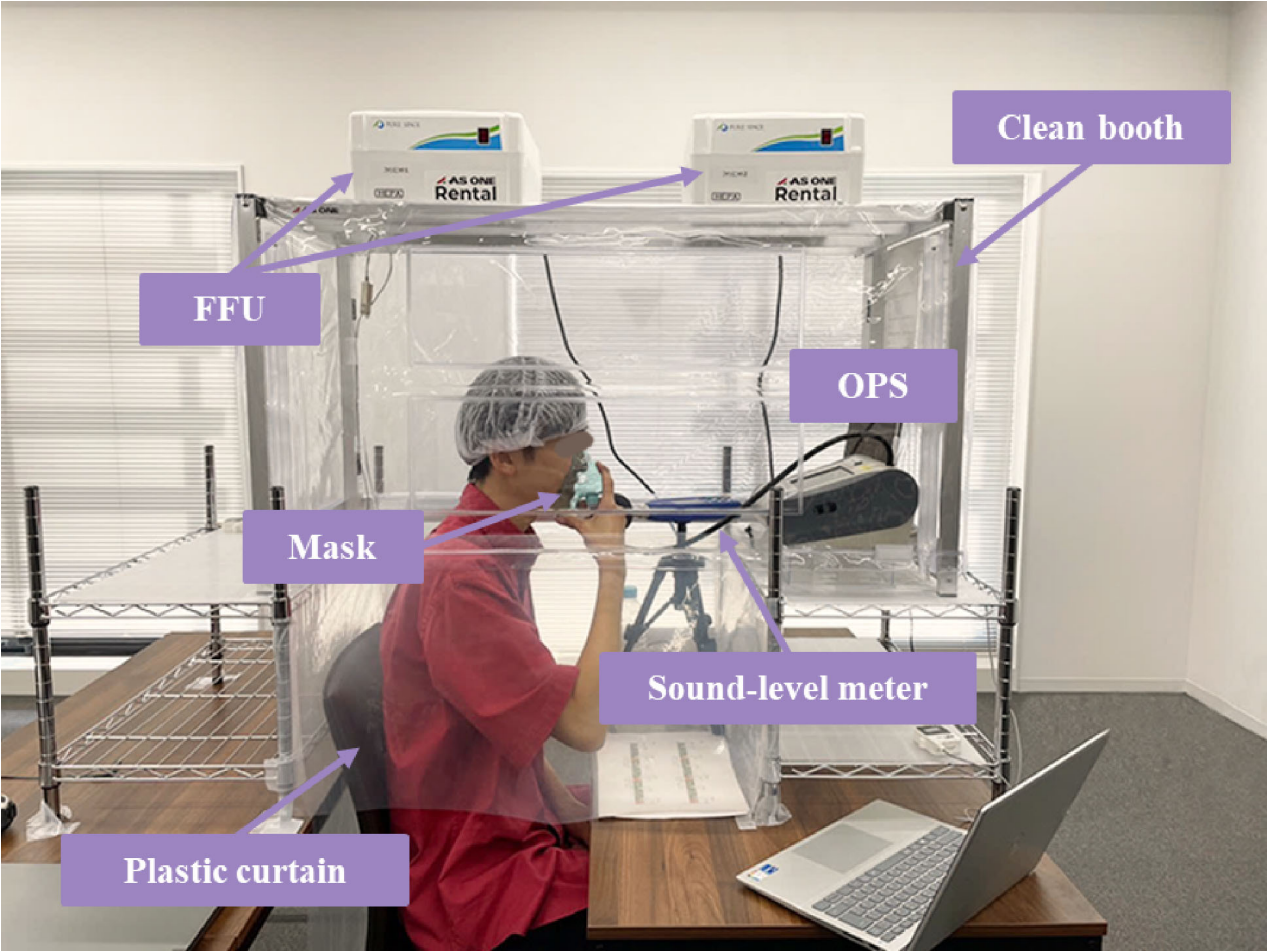
Experimental setup The clean booth was set up in the laboratory and OPS, and a sound-level meter was installed. Participants sat with their heads inside the clean booth and timed their speech by looking at a clock placed next to the OPS.

The experimental setup is shown in Fig. 1. To minimize the background effects of contaminants in the air, a clean booth (AS ONE: CB1000) was installed in the laboratory where the experiments were performed. The clean booth was placed on a stand at a height that allowed people to enter. The gap resulting from this specification was covered with a plastic curtain to prevent indoor air from entering the clean booth. The volume of the clean booth, including the area covered with plastic curtains, was 0.38 m^3^, and ventilation was provided at 120 m^3^/h (33.3 L/s) through two fan filter units (FFU) with high efficiency particulate air (HEPA) filters. Preliminary experiments confirmed the effect of the distance between the mouth and the sound-level meter on sound pressure, with the sound-level meter placed approximately 10 cm from the mouth.

**Fig. 2:**
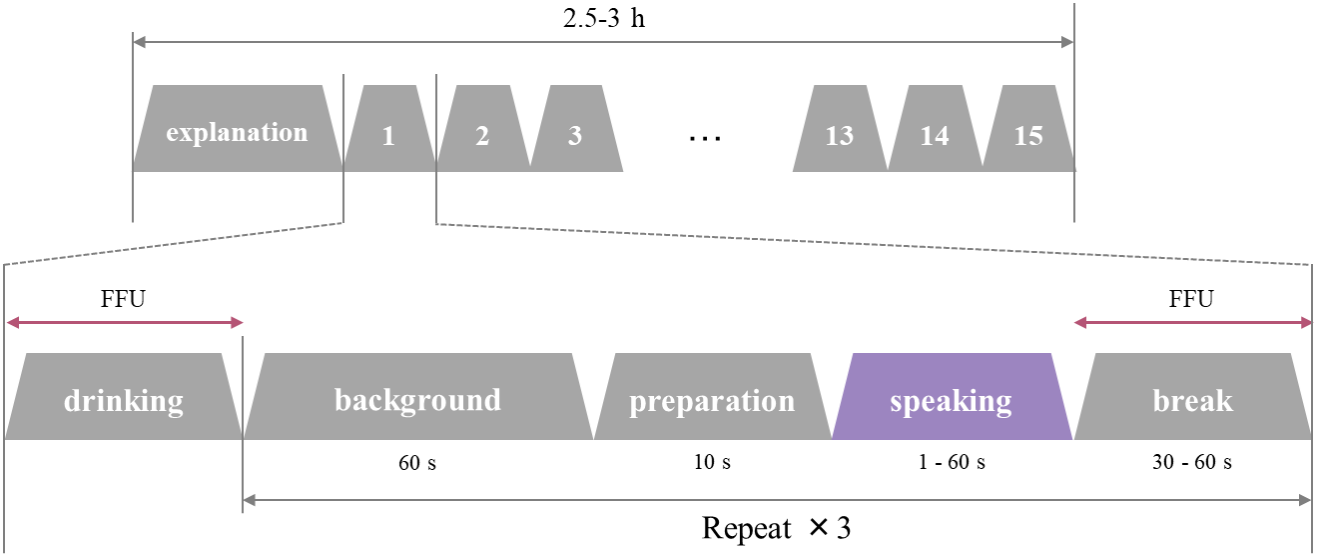
Flow of the experiment The procedure was initiated with the participant drinking water and then measuring the background concentration for 60 s. Next, they prepared to hold the mask for 10 s before starting their speech. There was a short break following the speech, after which the speech was repeated. This sequence was performed three times for each pattern. With a total of 15 patterns, the entire process took approximately 2.5–3 hours per participant.

As shown in Fig. 2, after a brief description of the experiment, speech experiments were conducted in the order shown in Table 1, from pattern 1 to 15. Participants were asked to drink water to adjust their oral condition before starting each pattern. The background concentration and speech measurements were repeated three times, and the experiment took approximately 2.5–3 hours per participant.

The operating noise of the FFU in the clean booth was 60–70 dB, which affected voice volume measurement. Thus, the FFU was stopped during the background concentration measurements and speech. During the breaks, the FFU was operated for approximately one minute to clean the environment inside the booth.

If the participants became blocked in their speech or made reading errors, the FFU was used to clean the air environment. The participants were then asked to repeat the same speech pattern.

This study targeted the particles generated through respiratory activities, including speech, for measurement. The OPS was placed in front of the face, as shown in Fig. 1, and it was necessary to ensure that the participants’ exhalation was not measured by the measuring section of the OPS during breaks. Therefore, during background concentration measurement, the participants were seated and asked to turn their faces to one side. Hence, they breathed quietly such that the aerosol particles generated by breathing did not affect the measurement. The concentration of the particles generated from the participants’ bodies and from building materials was used as the background concentration.

### Participants and selection criteria

We conducted an open recruitment process within the university. Inclusion criteria comprised: (i) being in good health, (ii) being able to speak normally, and (iii) being able to speak Japanese fluently. No other restrictions were imposed, and individual physique, throat characteristics, throat fatigue, and lung capacity were not considered.

### Speech sentences

Based on the study by Asadi et al. [7] previously reported a correlation between phoneme type and particle emission, we modified the emergency discourse in the early stages of the COVID-19 outbreak [10] through focusing on the proportion of vowels and voiceless fricatives. As shown in Additional file 2, Japanese vowels involved five tones (a, i, u, e, o) and voiceless fricatives comprised 18 tones (phonemes composed of /f/, /h/, /s/, and /ʃ/ and vowels combinations). Additional file 3 shows information regarding the phonemes in the sentences, and Additional file 4 shows the Japanese sentences used in the experiment, written in alphabetic characters. However, the phonemes were not adjusted for the 1s phrase “Ohayou gozaimasu” (“good morning” in Japanese) as this is a common greeting in Japan.

### Calculation of aerosol particle concentration

The aerosol particle data measured using the OPS were output as number concentrations. The number concentration refers to the number of particles contained per unit volume. We measured the background concentrations for 1 min and subtracted the mean of the background concentration from the number concentration measured during speech, which was treated as the result. The mass concentration refers to the total mass of particles contained per unit volume and was calculated through multiplying the number concentration by the volume of aerosol particles and density of water. The volume of aerosol particles was calculated for each particle size range using the median value of each particle size range as the particle diameter. In this case, the mass concentration represents the total mass of the particles per unit volume and is influenced by the mass of relatively large particles. Specifically, 80–90% of aerosol particles larger than 5.0 µm were deposited in the nasopharynx of the upper respiratory tract, whereas some particles smaller than 5.0 µm in size reached the alveoli of the lower respiratory tract [11, 12]. This study used two indicators, namely, number concentration and mass concentration, to analyze the effect of both relatively large and small particles emitted during speech.

The conductive silicone tube extending from the mouth to the OPS measurement section was approximately 60 cm long with an internal diameter of 5 mm. The analysis took into account a 0.35 s delay as the aerosol particles moved through the tube.

### Statistical analysis

To perform a significance test for aerosol particle concentrations in the five speech duration patterns for each voice volume, a Friedman test was performed using IBM SPSS Statistics Ver. 28 software. In tables, a significance level of *p* < 0.1 is indicated by ‘†’, *p* < 0.01 by ‘**’, and *p* < 0.001 by ‘***’. Additionally, a Wilcoxon signed-rank test was performed between the two groups to calculate the effect size, *r* [13]. Effect size, a measure that is independent of sample size and expresses the strength of the relationship between variables, was analyzed as recommended by the American Psychological Association [14].

## Results

### Voice volume distribution

**Fig. 3:**
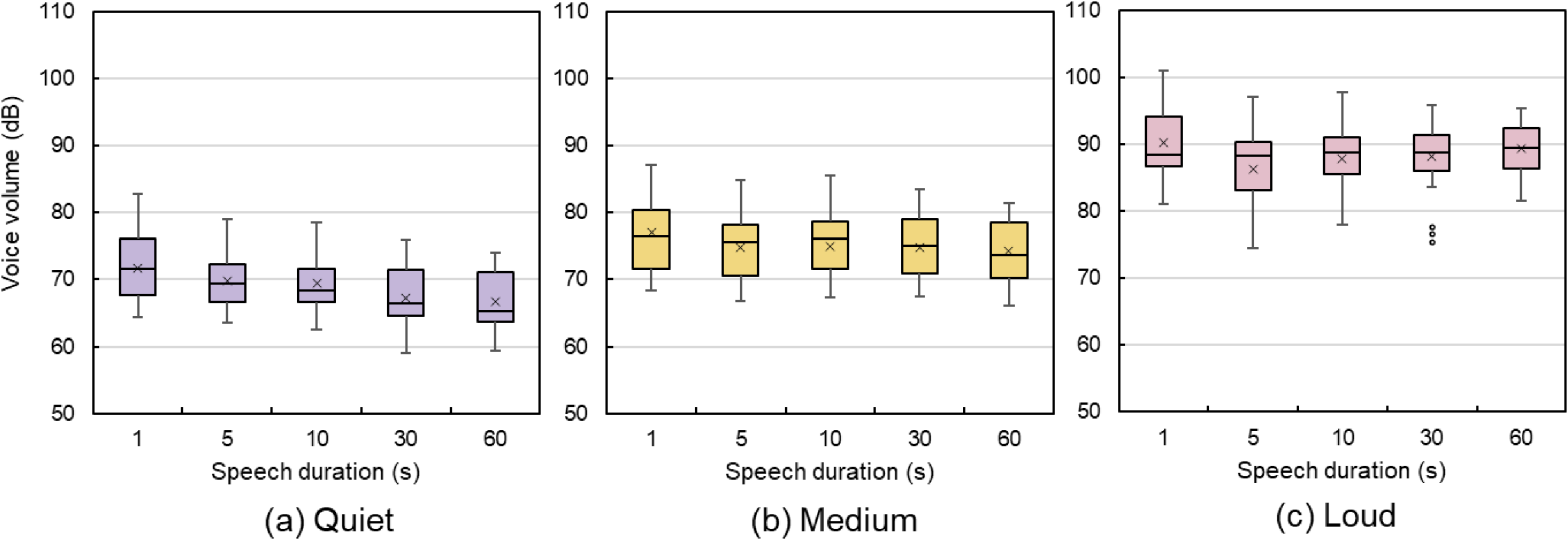
Voice volume distribution The graphs show the range of voice volumes for each pattern. Individual differences in voice volume significantly affect voice volume, with one participant’s quiet voice volume sometimes becoming another’s medium voice volume. (a) The quiet voice volume range was 59.1–82.9 dB. (b) The medium volume range was 66.2–89.6 dB. (c) The loud volume range was 74.6–103.7 dB. In the figures, the mean values are shown with ×. Other graphs presented in this paper are shown in the same way. Outliers are also indicated separately by different symbols for each participant.

The equivalent noise level was calculated for each speech duration and the value obtained was used as the voice volume (Fig. 3) [15]. Quiet volume ranged from 59.1 dB to 82.9 dB, medium volume ranged from 66.2 dB to 89.6 dB, and loud volume ranged from 74.6 dB to 103.7 dB. Within the same voice volume, the voice volume for 1 s speech was higher. Individual differences in voice volume significantly affected the voice volume, with one participant’s quiet voice volume sometimes becoming another’s medium volume. However, when assessing the risk of infection, it is important not to standardize the sound level but to focus on the loudness of the voice that is normally used. Therefore, in this study, voice volume was not classified based on the equivalent noise level but analyzed in terms of loud, medium, and quiet categories.

### Particle size distribution

**Fig. 4:**
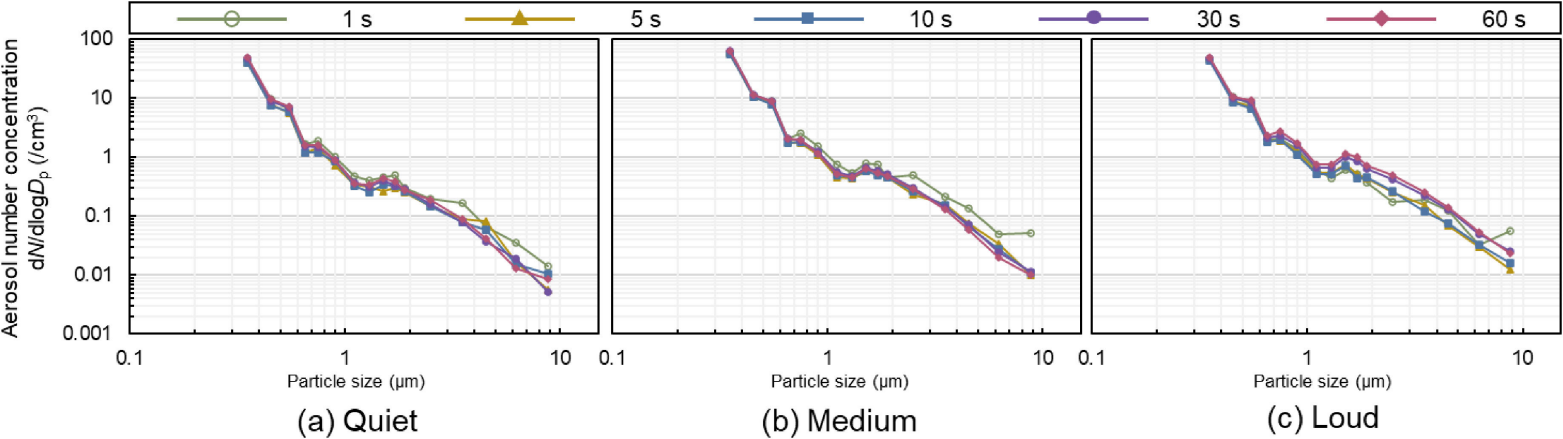
Particle size distribution of the aerosol number concentration This figure shows the particle size distribution in aerosol number concentration for five speech patterns for each voice volume. The larger the particle size, the lower the concentration for all speech durations.

**Fig. 5:**
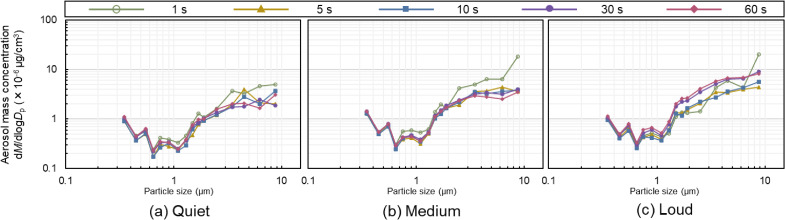
Particle size distribution of the aerosol mass concentration This figure shows the particle size distribution in the aerosol mass concentration of the five speech patterns for each voice volume. The larger the particle size, the greater the concentration for all speech durations. For all speech durations, the particle size distribution for the 1 s speech duration is more varied than for the other speech durations.

Figures 4 and 5 show the particle size distributions of aerosol number and mass concentration, respectively. The average aerosol particle concentrations for the 11 participants were calculated for each speech duration and are presented for each particle size. In Fig. 4, the distribution is such that the number concentration is high for small particles and low for large particles. In the (a) quiet and (b) medium conditions, the difference in speech duration was small. However, in the (c) loud condition, approximately 0.6–1.0 µm in particle size, the number concentration at 30 and 60 s was higher than that at 5 s and 10 s. There were also four peaks in the particle size distribution regardless of the speech duration: 0.3–0.4 µm, 0.5–0.6 µm, 0.7–0.8 µm, and 1.4–1.6 µm. The same trend can be observed for the mass concentrations in Fig. 5 with respect to the number concentrations. Figure 5 shows the distribution of higher aerosol mass concentrations for larger particle sizes. For the 1 s speech, there was a variation in the particle size distribution, as shown in Figs. 4 and 5.

### Respiratory aerosol particle concentration

**Fig. 6:**
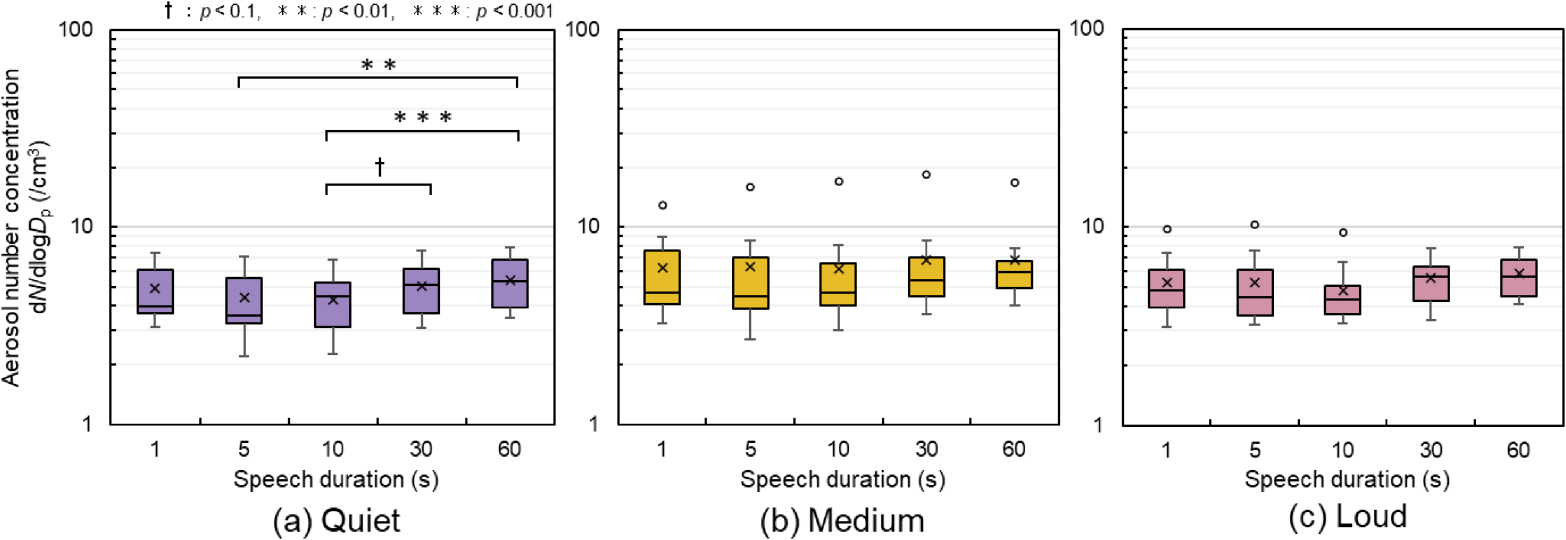
Aerosol number concentration for each pattern (a) Quiet voice volume: the concentration was significantly higher at 60 s of speech than that at 5 and 10 s of speech. There was also a significant increasing trend for concentration at 30 s when compared with 10 s. (b) Medium voice volume: the median value tended to increase from 5 to 60 s of speech. Outliers (Participant A, indicated by ○) were identified in all patterns, but no significant differences were observed. (c) Loud voice volume: outliers (Participant A) were identified at 1, 5, and 10 s of speech. †, *p* < 0.1; **, *p* < 0.01; ***, *p* < 0.001

**Fig. 7:**
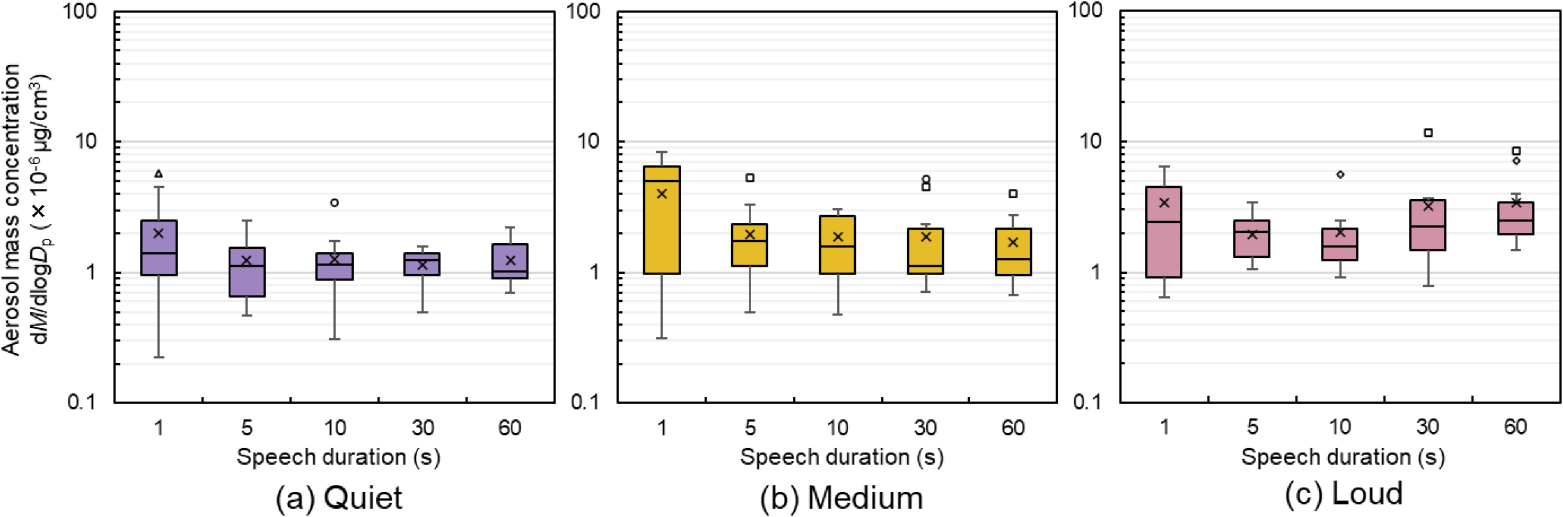
Aerosol mass concentration for each pattern (a) Quiet voice volume: minimal trend observed. (b) Medium voice volume: the inter-quartile range in 1 s speech was larger than those in others. (c) Loud voice volume: the median value tended to increase from 10 to 60 s of speech. A total of four participants (indicated separately using different symbols) recorded outliers. Participant B (indicated by □) recorded outliers in five of the 15 patterns.

Figures 6 and 7 show the aerosol particle concentrations under each condition. The aerosol particle concentrations shown refer to the time-averaged aerosol particle concentrations measured during speech for each condition in each speech pattern, and represent the values for the 11 participants.

As shown in Fig. 6, the number concentration at 60 s was significantly higher for quiet voice volumes than at 5 s and 10 s. There was also a significant trend in number concentration for 30 s speech compared with 10 s speech. No significant differences were observed in loud and medium voice volumes, but a rising trend was noted in the median values for 30 and 60 s compared with 5 and 10 s of speech. Effect sizes were analyzed using a three-level indicator: small difference, *r* > 0.1; moderate difference, *r* > 0.3; and large difference, *r* > 0.5. The results indicated that as *r* increased the likelihood of significance also increased with an increasing number of participants. As shown in Table 2, large differences were observed between 10 s and 30 s and between 10 s and 60 s at medium volume, and across speech patterns exceeding 10 s at loud volume. Notably, eight of the 15 patterns showed outliers, all from the same participant, namely, Participant A, who is a female participant. On average, Participant A recorded 2.30 times above the median number concentration. Specifically, during a medium-volume, 10 s speech, Participant A’s concentration was 3.67 times above the median.

**Table 2:** Comparison of effect sizes between each speech patterns at aerosol number concentrations

	**Effect size *r* (-)**				
	**1 s**	**5 s**	**10 s**	**30 s**	**60 s**
**Quiet**					

1 s					
5 s	**0.617**				
10 s	**0.590**	0.161			
30 s	0.188	**0.643**	**0.885**		
60 s	**0.509**	**0.751**	**0.831**	**0.643**	

**Medium**					

1 s					
5 s	0.107				
10 s	0.375	0.134			
30 s	0.322	0.429	**0.670**		
60 s	0.295	0.429	**0.590**	0.027	

**Loud**					

1 s					
5 s	0.080				
10 s	**0.670**	0.295			
30 s	0.214	0.107	**0.697**		
60 s	0.483	0.375	**0.777**	**0.751**	

Small difference, *r* > 0.1; moderate difference, *r* > 0.3; large difference, *r* > 0.5

Analysis is based on a three-tier system: small difference for *r* > 0.1, moderate difference for *r* > 0.3, and large difference for *r* > 0.5. Large differences are indicated in bold. This table compares the effect sizes of aerosol number concentrations across the two speech patterns. Large differences were observed in medium voice volumes between 10 and 30 s, between 10 and 60 s, and in loud voice volumes across speech patterns exceeding 10 s.

As shown in Fig. 7, no significant differences were observed in mass concentrations; however, an upward trend in the median values was apparent from 10 s to 60 s for loud voice volumes. Table 3 shows large differences in effect sizes for loud voice volumes between 5 s and 60 s, 10 s and 30 s, and 10 s and 60 s. In contrast, between 1 s and the other four sentence patterns, greater than moderate differences were identified for the quiet volume and large differences were identified for the medium volume. Four participants recorded outliers in mass concentration, including Participant A. Of the four participants, Participant A recorded mass-concentration outliers in two of the 15 patterns. Participant B, who is also female participant, recorded outliers in five of 15 patterns, with an average mass concentration of 2.21 times above the median. Notably, during a loud-volume, 30 s speech, Participant B’s concentration reached 5.24 times above the median.

**Table 3:** Comparison of effect sizes between each speech patterns at aerosol mass concentrations

	**Effect size *r* (-)**				
	**1 s**	**5 s**	**10 s**	**30 s**	**60 s**
**Quiet**					

1 s					
5 s	0.375				
10 s	**0.509**	0.000			
30 s	0.429	0.027	0.080		
60 s	0.322	0.080	0.000	0.134	

**Medium**					

1 s					
5 s	**0.509**				
10 s	**0.617**	0.134			
30 s	**0.509**	0.295	0.080		
60 s	**0.590**	0.241	0.214	0.107	

**Loud**					

1 s					
5 s	0.268				
10 s	0.295	0.134			
30 s	0.027	0.429	**0.563**		
60 s	0.027	**0.617**	**0.724**	0.268	

Small difference, *r* > 0.1; moderate difference, *r* > 0.3; large difference, *r* > 0.5

Table 3 uses the three levels of effect size indicators listed in Table 2. This table compares the effect sizes of the aerosol mass concentrations across the two speech patterns. Large differences were observed in loud voice volumes between 5 s and 60 s, 10 s and 30 s, and 10 s and 60 s. Between 1 s and the other four sentence patterns, more than moderate differences were observed for quiet volume and large differences for medium volume.

## Discussion

### Variation in particle size distribution

A difference was observed between the particle size distributions for short (5 s and 10 s) and long (30 s and 60 s) loud speech durations. The reason for the higher concentration of particles larger than 0.6–1.0 µm in diameter may be that saliva accumulates in the oral cavity during prolonged speech. Furthermore, more aerosol particles may be released during loud speech compared with quiet and medium-volume speech owing to the increased expiratory airflow and velocity in the respiratory tract.

There were four peaks in the particle size distribution in the study, regardless of the duration of speech: 0.3–0.4 µm, 0.5–0.6 µm, 0.7–0.8 µm, and 1.4–1.6 µm. The particle size distribution in the study by Morawska et al. [5] consisted of modes with peaks at 0.80 ± 0.08 µm, 1.8 ± 0.3 µm, 3.5 ± 0.7 µm, and 5.5 ± 1 µm. The peak distributions of the aerosol particle concentrations in this study differed from their results. A possible reason is the difference in distance from the mouth to the aerosol particle measurement section. They compared particle size by changing the distance from the mouth to the measurement section, showing that the size of the peaks decreased as the distance increased. While they conducted experiments at distances of 1 cm and 30 cm, this study was conducted at a distance of 60 cm, which may have contributed to the smaller peaks observed. Furthermore, the APS used in their study had a measured particle size range of 0.5–20 µm, while the OPS used in this study had a range of 0.3–10 µm, making a complete comparison impossible. Therefore, further analysis is needed in future studies to determine the effect of similar modes in particle size distribution.

### Relationship between speech duration and aerosol particle concentration

Figure 6 shows significant differences were observed only in quiet voice volumes. No significant differences were observed for medium and loud voices; however, the medians were higher for 30 s and 60 s than for 5 s and 10 s. In the analysis of the effect size shown in Table 2, large differences were observed for medium voice volumes between 10 s and 30 s and between 10 s and 60 s and in loud voice volumes for speech patterns longer than 10 s. Therefore, as the number of participants increased, an increasing trend in aerosol number concentration was also observed at medium and loud voice volumes. It is possible that more aerosol particles are released through the movement of the vocal cords, lips, and tongue between phonemes in a sentence rather than only during the utterance of phonemes, as hypothesized. Saliva may accumulate in the oral cavity over an extended period. While individual differences may exist, it has been suggested that voice pitch, inflection, and breathing timing are related to the aerosol particle concentration.

Figure 7 indicates that the range of the measured aerosol mass concentration for 1 s speech is wider than that for other speech durations. A potential reason is that in the 1 s speech sentence “Ohayou gozaimasu,” the proportion of vowels and voiceless fricatives is not adjusted, which differs from the proportion of phonemes in the other sentences. Additionally, a 1 s speech makes it difficult for participants to adjust their voice volume, and the average equivalent noise level for a 1 s speech is louder than that for other speech durations, as shown in Fig. 3. Therefore, voice volume adjustments are important when comparing short and long speech durations.

### Duration of respiratory activity

Our findings suggest that aerosol number concentration increases with longer speech duration. Consequently, incorporating speech duration into concentration assessments can enhance the evaluation of the risk of infection. Relying on uniform concentration for both short and long speech durations might lead to underestimating the risks associated with longer speech. In the case of mass infection during choral singing [16], the act of choral singing, which involves loud and prolonged speech, may increase the concentration of aerosol particles more easily than normal, thus creating a more favorable environment for infection. In restaurants, conversations generally occur at seating positions. In addition to long conversations occurring in close proximity, voices naturally become louder to avoid disturbance by those around them. Therefore, in relation to a restaurant-related COVID-19 outbreak in Guangzhou, China [17], prolonged speech may have played a role in addition to ventilation issues. Therefore, research linking aerosol particle concentrations to the duration of respiratory activities, including speaking, is essential for accurate infection risk assessment during such activities.

### The existence of super-emitters

In this study, Participant A and Participant B recorded statistical outliers in more than five patterns and exhibited higher aerosol particle concentrations than the average. This finding aligns with those of Asadi et al. [6] and Ahmed et al. [9], who discussed super-emitters. Our study defined a super-emitter as a participant who recorded statistical outlier in five or more of 15 speech patterns based on the definition by Ahmed et al. [9]. Asadi et al.’s experiments [6] showed maximum aerosol particle concentrations 3.02 times above the median when participants vocalized the sound /a/ for 5 s, and 5.60 times above the median when reading an English passage. Ahmed et al. [9] observed a maximum concentration of 3.43 times above the median when participants spoke the sound /a/ for 5 s, similar to Asadi et al. Comparing these results, Participant A could be considered a super-emitter in terms of number concentration, emitting numerous small particles, and Participant B, in terms of mass concentration, emitting larger particles. However, no significant difference was observed between the particle sizes emitted by the super-emitters. Participant A recorded outlier values in two patterns for mass and number concentrations. Therefore, it can be assumed that both particle sizes were released without distinction. Thus, it appears that being a super-emitter may be independent of particle size. Their particle size distribution and the average particle size distribution of the other participants (n = 10) are shown in Additional Files 5 and 6.

### Limitations

This study was limited by the experimental period, the small number of participants, and the capabilities of the experimental equipment. Each participant performed the same speech pattern three times over 2.5–3 hours due to the constraints of the experimental period. However, conducting the experiment on separate days is considered effective in reducing participant fatigue. Future studies should include a larger number of participants and employ more precise equipment capable of measuring a broader spectrum of particle sizes. It is crucial to conduct experiments in a clean environment with a low background concentration. Moreover, our focus on speech duration constrained the analysis of super-emitters. The investigation of super-spreading phenomena, potentially involving super-emitters, is essential for understanding transmission dynamics. In this study, due to ethical review considerations, we were unable to conduct a detailed analysis of the physical characteristics of super-emitters. Therefore, further research is necessary to explore these aspects, including detailed experiments investigating the relationship between voice frequency and physical traits such as throat structure.

## Conclusions

To explore the effects of speech duration and voice volume on respiratory aerosol particle concentrations, we experimented with participants performing 15 speech patterns across five durations and three volumes. Voice volumes and aerosol particle concentrations were measured during these speeches. Analysis of data from 11 participants indicated that over speech durations ranging from 10 s to 60 s, the aerosol particle concentration generally increased. Considering speech duration may provide a better understanding of the dynamics of aerosol generation. Therefore, studies considering extended speech durations rather than only using short durations are needed. According to our analysis in terms of potential super-emitters, two participants were identified as possible super-emitters.

## Abbreviations

APS: aerodynamic particle sizer
FFU: fan filter unit
HEPA: high efficiency particulate air
OPS: optical particle sizer
s: seconds

## Supplementary information

**Additional file 1:** **Supplementary Table 1** Particle size range of the OPS. **Additional file 2:** **Supplementary Table 2** Phonemes in Japanese. **Additional file 3:** **Supplementary Table 3** Information on phonemes in speech sentences. **Additional file 4:** **Supplementary Table 4** Sentences (Japanese in the alphabet and English translation). **Additional file 5:** **Supplementary Fig. 1** Particle size distribution of aerosol number concentration for Participant A. **Additional file 6:** **Supplementary Fig. 2** Particle size distribution of aerosol mass concentration for Participant B.

## Declarations

### Availability of data and material

The datasets of this paper are available from the corresponding author upon reasonable request.
