# Supplementary material for: Effects of speech duration and voice volume on the respiratory aerosol particle concentration
Source: Environ Health Prev Med. 2025 Mar 5;30:14. doi: 10.1265/ehpm.24-00251 (PMC11925707; doi:10.1265/ehpm.24-00251)
Supplement: Supplementary file 1 — Additional file 1: Supplementary Table 1 Particle size range of the OPS. Additional file 2: Supplementary Table 2 Phonemes in Japanese. Additional file 3: Supplementary Table 3 Information on phonemes in speech sentences. Additional file 4: Supplementary Table 4 Sentences (Japanese in the alphabet and English translation). Additional file 5: Supplementary Fig. 1 Particle size distribution of aerosol number concentration for Participant A. Additional file 6: Supplementary Fig. 2 Particle size distribution of aerosol mass concentration for Participant B. [file ehpm-30-014-s001.docx]

Effects of speech duration and voice volume on the respiratory aerosol particle concentration

**Additional files (n = 6)**

**Additional file 1: Supplementary Table 1** Particle size range of the OPS

| **Particle Size Range** | **Ch.1** | **Ch.2** | **Ch.3** | **Ch.4** | **Ch.5** | **Ch.6** | **Ch.7** | **Ch.8** |
| --- | --- | --- | --- | --- | --- | --- | --- | --- |
| Lower limit (µm) | 0.3 | 0.4 | 0.5 | 0.6 | 0.7 | 0.8 | 1 | 1.2 |
|  | - | - | - | - | - | - | - | - |
| Upper limit (µm) | 0.4 | 0.5 | 0.6 | 0.7 | 0.8 | 1 | 1.2 | 1.4 |
|  |  |  |  |  |  |  |  |  |
| **Particle Size Range** | **Ch.9** | **Ch.10** | **Ch.11** | **Ch.12** | **Ch.13** | **Ch.14** | **Ch.15** | **Ch.16** |
| Lower limit (µm) | 1.4 | 1.6 | 1.8 | 2.0 | 3.0 | 4.0 | 5.0 | 7.5 |
|  | - | - | - | - | - | - | - | - |
| Upper limit (µm) | 1.6 | 1.8 | 2.0 | 3.0 | 4.0 | 5.0 | 7.5 | 10 |

The particle size measurement range of the OPS can be freely divided into 16 channels. As shown in the table, the channels were spaced at 0.1µm intervals up to 0.8µm, at 0.2µm intervals up to 2.0µm, at 1.0µm intervals up to 5µm, and at 2.5µm intervals up to 10.0µm.

**Additional file 2: Supplementary Table 2** Phonemes in Japanese

|  | **English phonetic Symbols** | **Japanese in Alphabet** | **Number of Phonemes** |
| --- | --- | --- | --- |
| Vowels | /a/, /e/, /i/, /o/, /u/ | a, i, u, e, o | 5 |
| Voiceless Fricatives | /f/, /h/, /s/, /ʃ/ | fa, fi, fe, fo, ha, hi, hu, he, ho sa, shi, su, se, so sha, shu, she, sho | 18 |

In Japanese, certain vowels and voiceless fricatives have been replaced by their English equivalents. As in English, vowels in Japanese consist of five letters: a, i, u, e, and o. The voiceless fricatives in Japanese include /f/, /h/, /s/, /ʃ/, along with various vowel combinations. The table identifies 18 specific sounds categorized as voiceless fricatives. The proportions of the phonemes in the sentences were adjusted to focus on vowels and voiceless fricatives.

**Additional file 3: Supplementary Table 3** Information on phonemes in speech sentences

| **Speech Duration (s)** | **Number of Japanese letters** | **Number of Phonemes** | | **Proportion (%)** | |
| --- | --- | --- | --- | --- | --- |
|  |  | **Vowels** | **Voiceless Fricatives** | **Vowels** | **Voiceless Fricatives** |
| 1 | 9 | 3 | 2 | 33.3 | 22.2 |
| 5 | 48 | 7 | 7 | 14.6 | 14.6 |
| 10 | 89 | 13 | 13 | 14.6 | 14.6 |
| 30 | 247 | 37 | 35 | 15 | 14.2 |
| 60 | 489 | 74 | 71 | 15.1 | 14.5 |

This table shows the number of Japanese letters, vowels, and voiceless fricatives and their respective proportions for each speech duration. We standardized the proportions of vowels and voiceless fricatives as much as possible. Except for 1 s, the percentages of vowels and voiceless fricatives ranged from 14.6% to 15.1% and 14.2% to 14.6% respectively. The phonemes of the 1-s sentence were not adjusted.

**Additional file 4: Supplementary Table 4** Sentences (Japanese in the alphabet and English translation).

| **Speech Duration (s)** | | | | **Sentences** | |
| --- | --- | --- | --- | --- | --- |
| 1s | | | | Japanese | Ohayou gozaimasu. |
|  |  |  |  | English | Good morning. |
| 60 | 30 | 10 | 5 | Japanese | Kankikaisuu towa, ichijikan ni heya ni torikomareru gaikiryou wo shitsuyouseki de watta mono wo sashimasu. |
|  |  |  |  | English | Ventilation rate is the amount of outside air drawn into the room per hour divided by the room volume. |
|  |  |  |  | Japanese | Kankihouhou niwa, mado wo akeru shizenkanki to, fuan wo riyousuru kikaikanki ga arimasu. |
|  |  |  |  | English | Types of ventilation include natural ventilation, by opening windows, and mechanical ventilation, by using fans. |
|  |  |  |  | Japanese | Mado ga aru tatemono ya norimono dewa, sekkyokutekini mado wo kaihoushite, gaiki wo torikomu houhou ga yuukoudesu. Jidousha dewa, naikijunkanmo-do ni suru no dewanaku, gaiki wo torikomu mo-do ni setteisuru koto ga yuukou desu. Fuan wo ugokashite kankisuru sai niwa, kyuuhaikikou ga shimatte inaika, fusagarete inaika wo, kakuninsuru koto ga hitsuyou ni narimasu. |
|  |  |  |  | English | In buildings and vehicles with windows, actively opening windows to let in outside air is an effective method. In vehicles, it is effective to set the mode to take in outside air rather than setting the mode to circulate inside air. When using fans for ventilation, check that the air supply and exhaust vents are not closed or blocked. |
|  |  |  |  | Japanese | Mado no nai heya demo, fuan wo setchisuru koto de, kankisuru koto ga kanou ni narimasu. Ofuisubiru nado dewa, kankikinou wo motsu kuuchousetsubi wo riyoushite, shitsunaikankyou ga tamotarete imasu. Ippanteki niwa, shouenerugi- wo kouryoshite, hitsuyouna kankiryou wo mitasu youni setsubi ga untensarete imasu. Taryouno gaiki wo torikomu to, reidamboukouritsu wa waruku natte shimaimasu ga, shitsunai deno gyoumu ni shishou ga dezu, zaishitsusha ga fuman wo motanai hani de, gaikiryou wo fuyasu nado no sochi wo toru koto wa, kanoudearu to suisoku saremasu. |
|  |  |  |  | English | Rooms without windows can also be ventilated by installing fans. In office buildings, the indoor environment is maintained by air conditioning systems with ventilation functions. In general, the systems are operated to provide the required amount of ventilation, taking into account energy savings. If large amounts of outside air are drawn in, the efficiency of the air conditioning system is reduced. However, it is assumed that it is possible to take measures such as increasing the amount of outdoor air to a level that does not interfere with indoor operation and does not cause discomfort to the occupants. |

Japanese sentences are transcribed using alphabetic characters. The participants spoke the “Japanese” sentences shown in the table. The 1-s sentence is “ohayou gozaimasu” (“good morning” in Japanese). The 60-s sentences were divided into sentences of 5 to 30 s (e.g., by reading all sentences written in 5-60 s, a 60-s sentence was formed). The English translations are also provided in the table.

**Additional file 5: Supplementary Fig. 1** Particle size distribution of aerosol number concentration for Participant A.


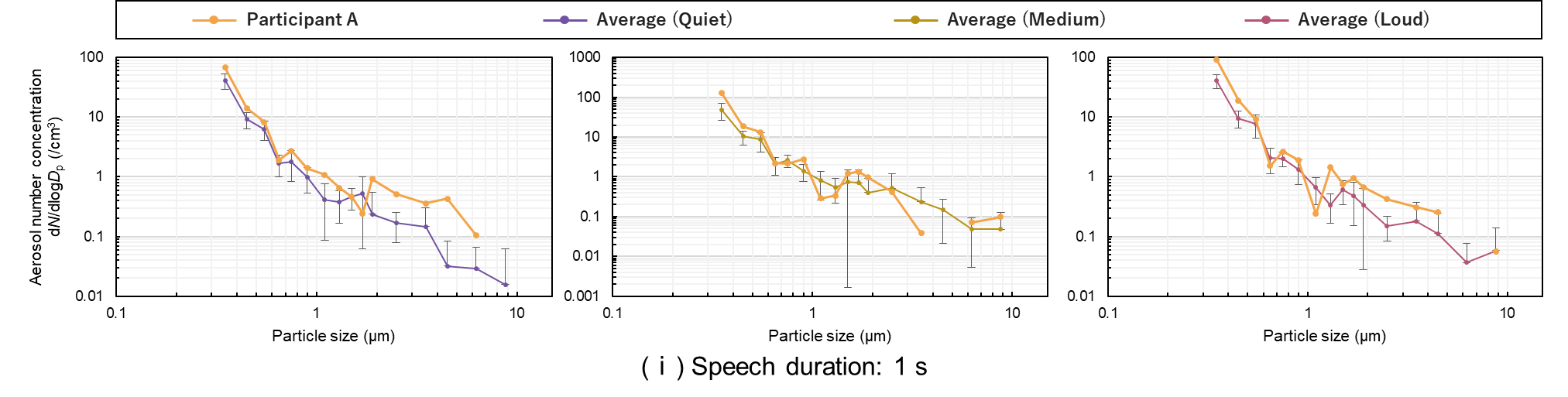

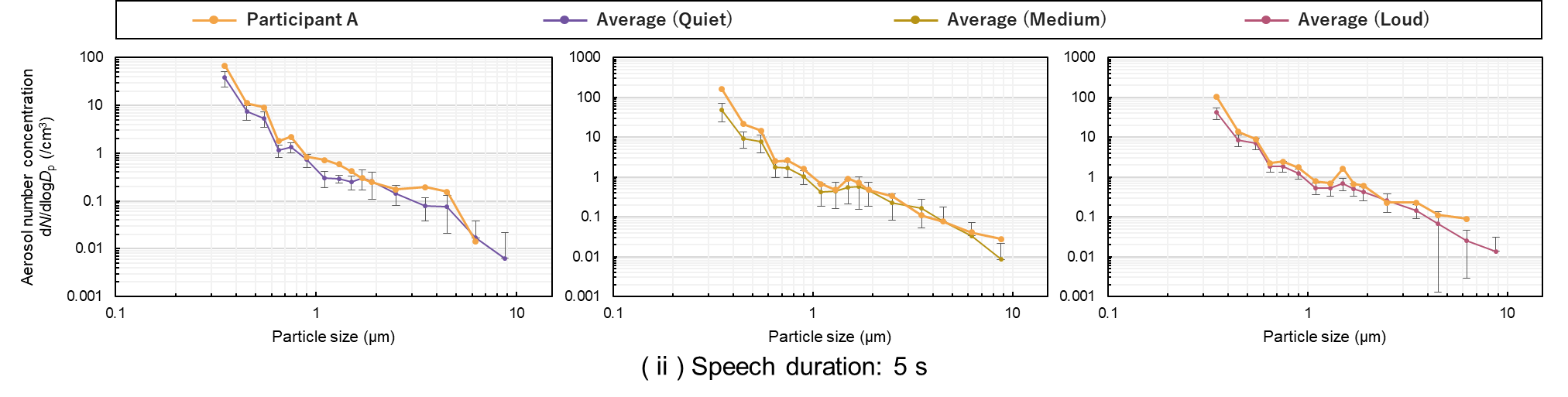

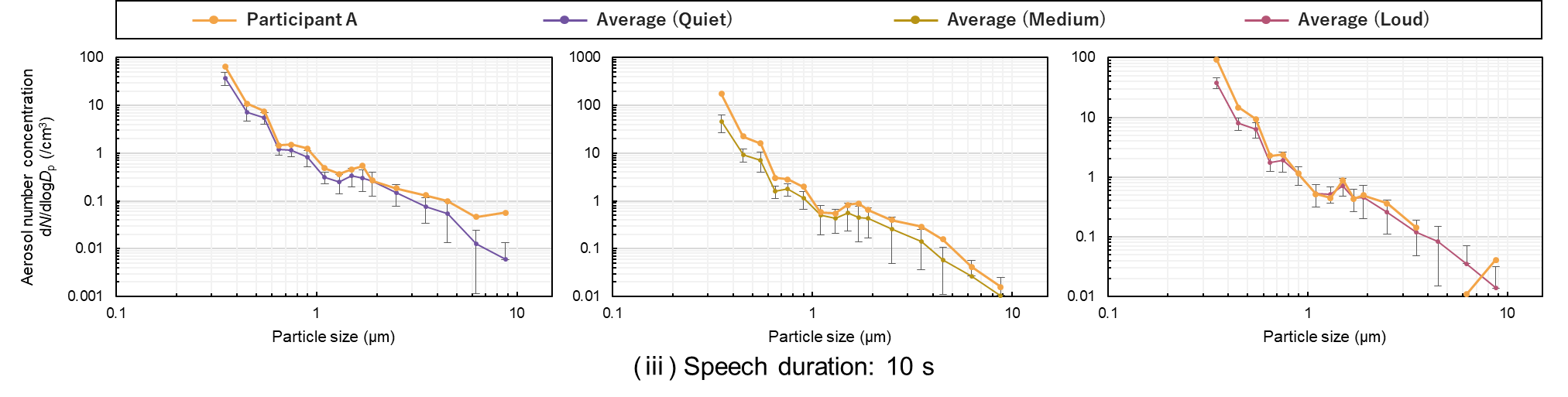

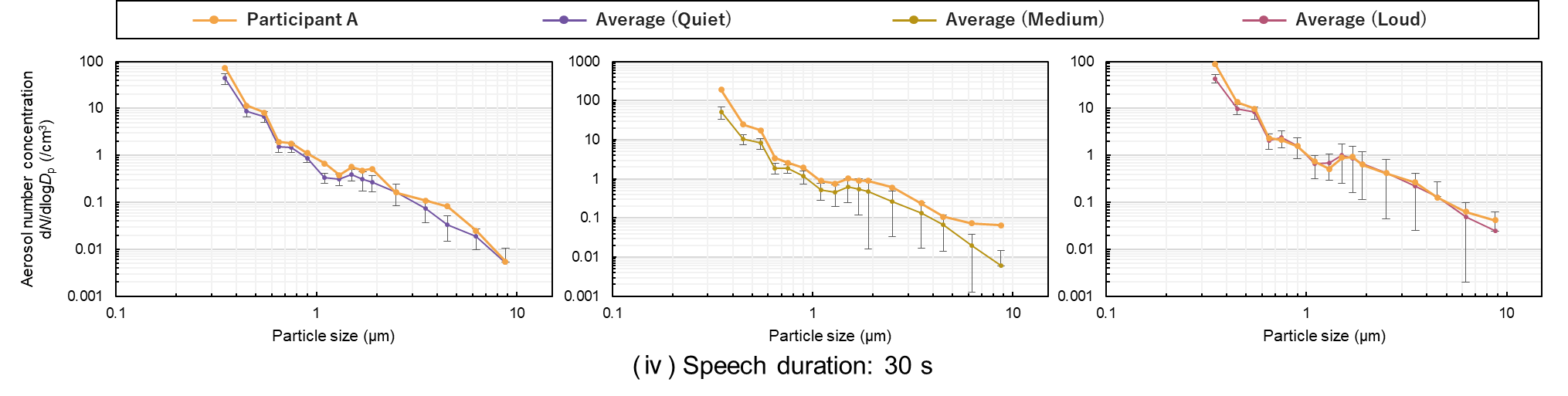

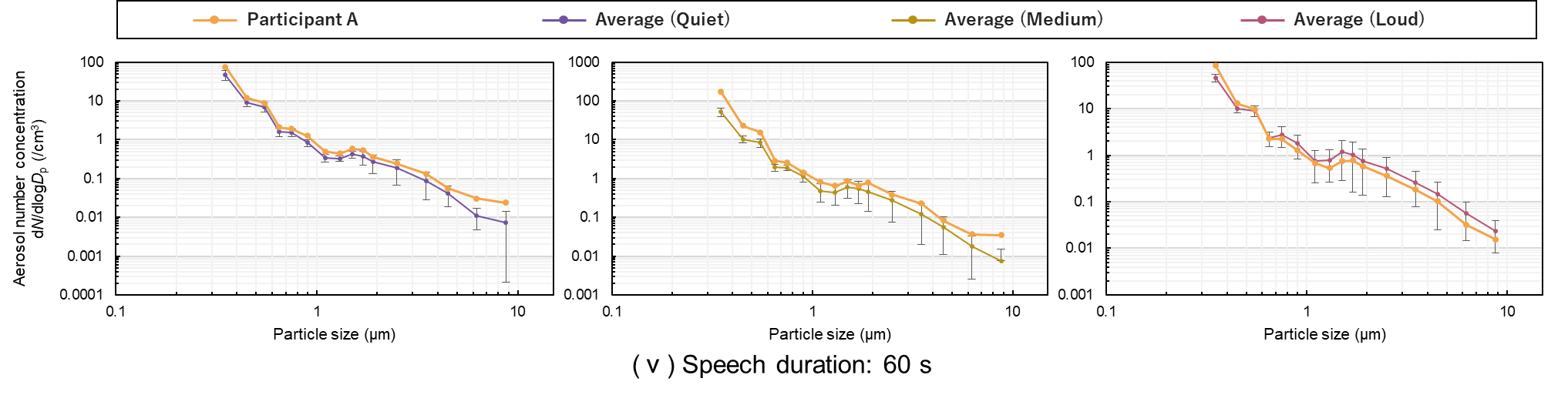


This graph illustrates the particle size distribution of number concentration across 15 speech patterns, showing the distribution for Participant A (a super-emitter) as well as the average distribution for the other participants (n=10). The average values for the 10 participants include error bars representing the standard deviation. In some cases, certain participants did not emit aerosol particles of specific sizes, resulting in an aerosol number concentration of zero.

**Additional file 6: Supplementary Fig. 2** Particle size distribution of aerosol mass concentration for Participant B.


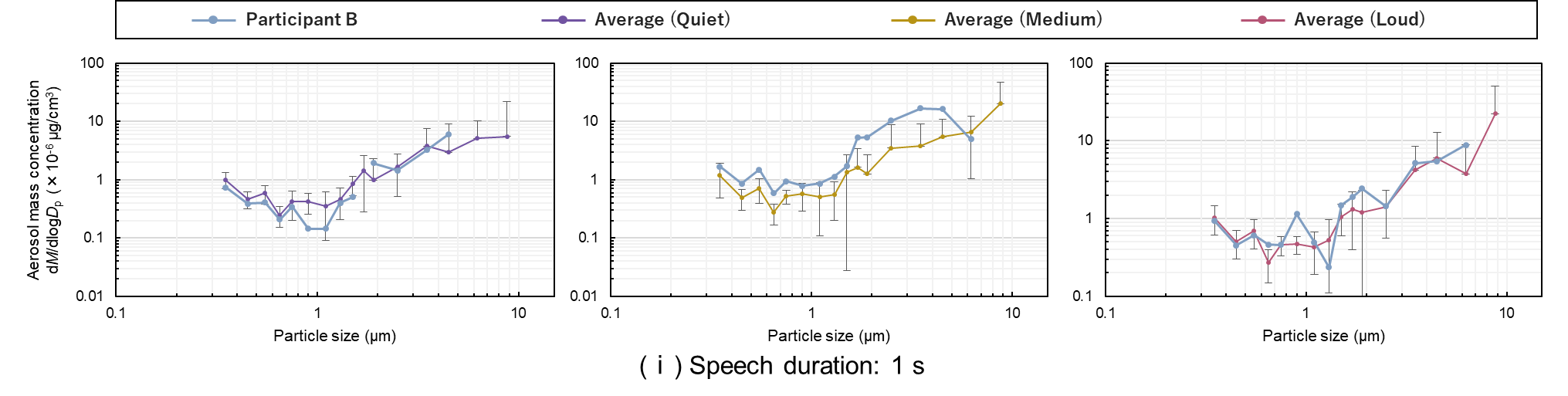

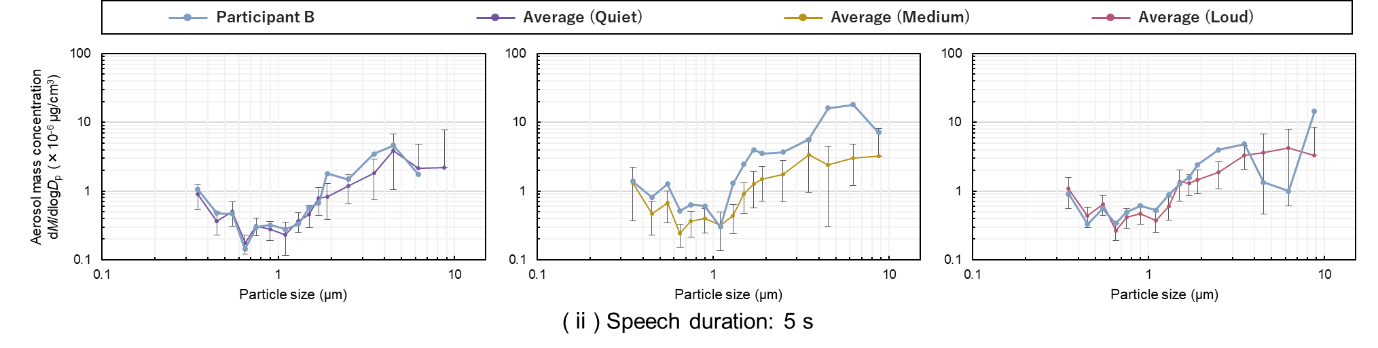

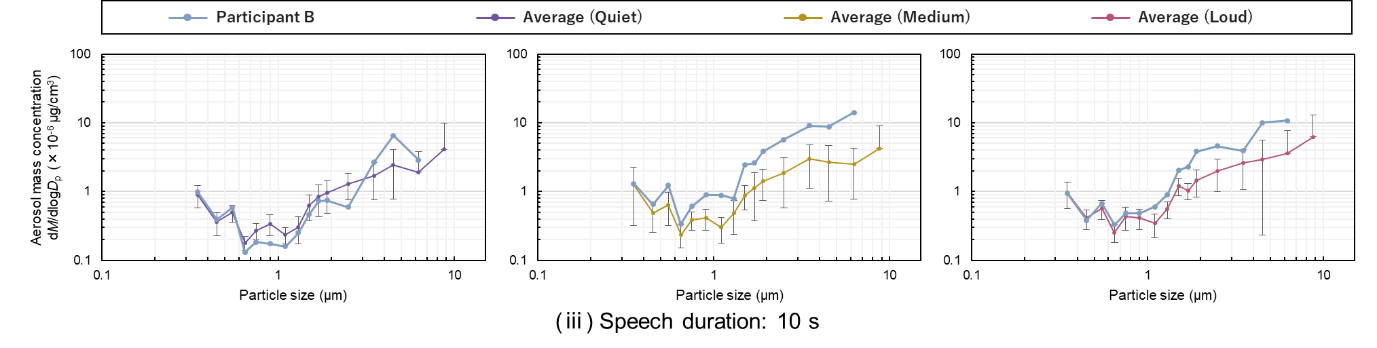

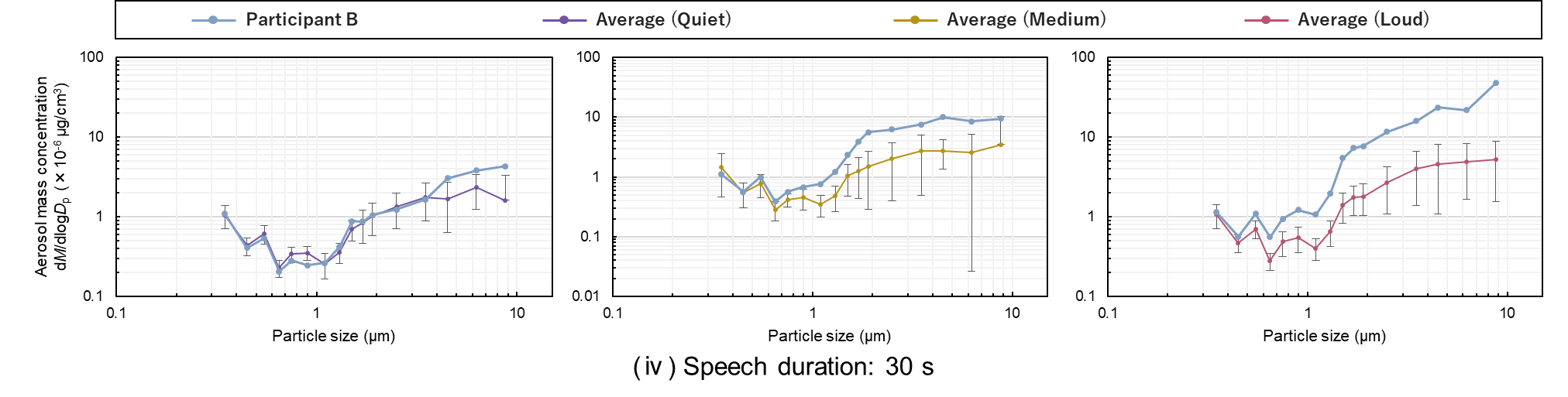

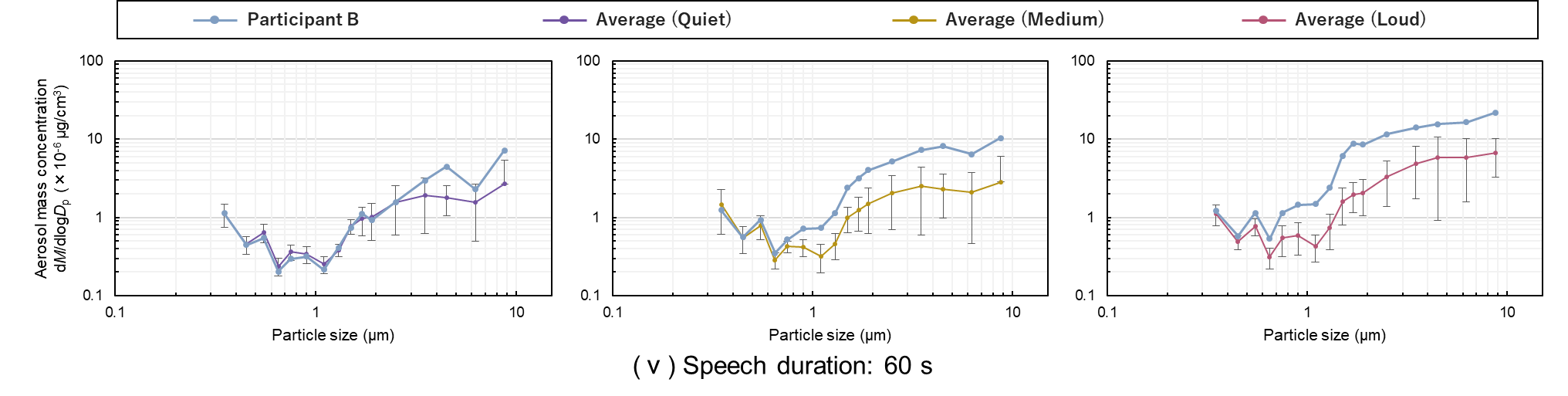


This graph illustrates the particle size distribution of mass concentration across 15 speech patterns, showing the distribution for Participant B (a super-emitter) as well as the average distribution for the other participants (n=10). Similar to Supplementary Fig. 1, the average values for the 10 participants include error bars representing the standard deviation. In some cases, certain participants did not emit aerosol particles of specific sizes, resulting in an aerosol number concentration of zero.
